# Supplementary figures and images for: Verification of emerging genomic mutations in Mycobacterium tuberculosis allows transmission chains to be distinguished in an epidemiological typing cluster extending over thirty years
Source: PLoS One. 2025 Jun 18;20(6):e0319630. doi: 10.1371/journal.pone.0319630 (PMC12176192; doi:10.1371/journal.pone.0319630)

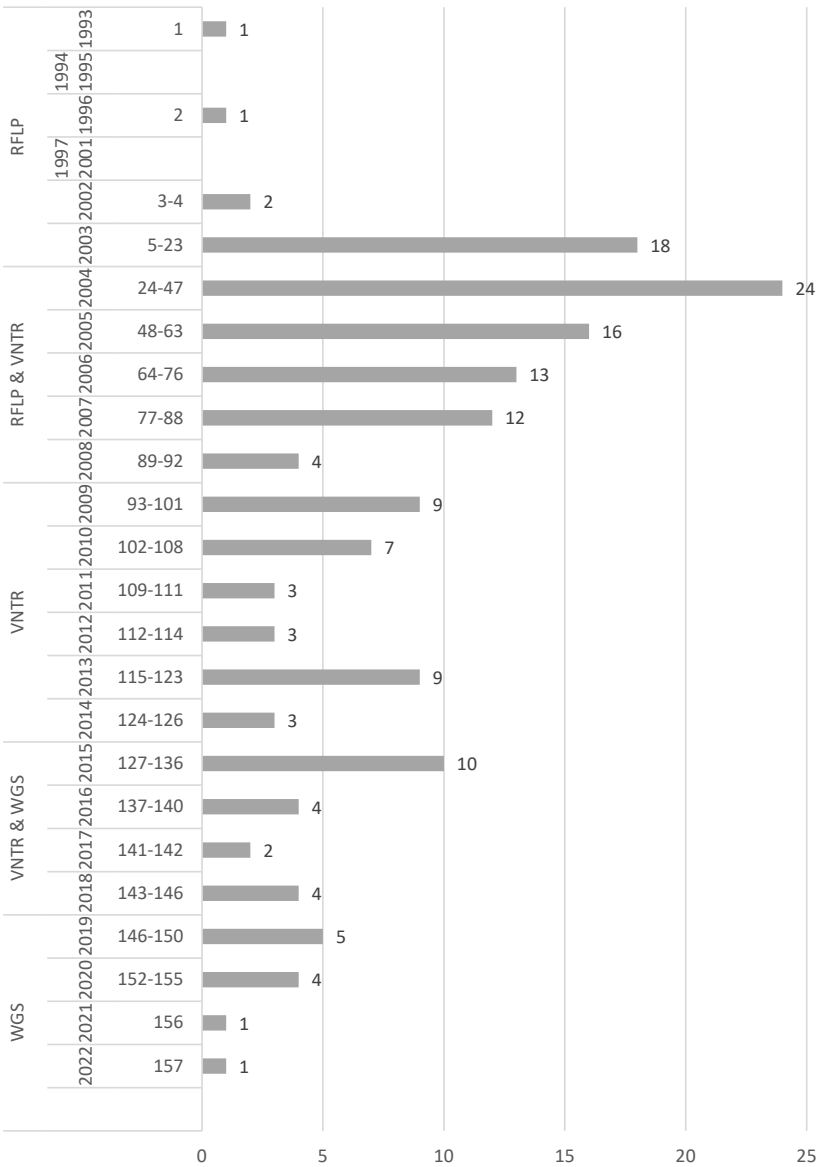

**Figure S1** Overview NTR numbers tb episode with typing method and year

Supplement: S1 Fig — (PDF) [file pone.0319630.s001.pdf]
